# Limited Access to Post-Acute Rehabilitation Care at Skilled Nursing Facilities with Medicaid

**DOI:** 10.1093/geroni/igaf122.3973

**Published:** 2025-12-31

**Authors:** Lilly Estenson, Erin Duffy, Jonathan Cloughesy, Mireille Jacobson

**Affiliations:** University of Southern California, Los Angeles, California, United States; University of Southern California, Los Angeles, California, United States; University of Southern California, Los Angeles, California, United States; University of Southern California, Los Angeles, California, United States

## Abstract

Medicaid beneficiaries often have trouble finding healthcare providers that accept their insurance due to Medicaid’s relatively low reimbursement rates. We conducted an audit study to examine Medicaid beneficiaries’ access to post-acute rehabilitation care at skilled nursing facilities (SNFs) in California, comparing SNF bed availability for patients with Medicare and Medicaid (“Medi-Medi”) to patients with Medicaid alone. We made calls between June and August 2025 to 968 SNFs listed as accepting Medicare and Medicaid in CMS Nursing Home Compare to inquire about bed availability for a fictitious grandparent being discharged from the hospital after hip fracture surgery. Patient insurance coverage was randomly assigned as Medi-Medi for calls to 483 SNFs and Medicaid alone for calls to 485 SNFs. Patient gender was randomized within each health insurance group. Callers did not specify patient age or additional health insurance details. Admission coordinators were reachable after up to five calls and able to provide information on bed availability at 72% of the SNFs. Among these SNFs, approximately 85% signaled bed availability and encouraged hospital transfer initiation for Medi-Medi patients. Coordinators were about 35 percentage points less likely to signal bed availability when patients had Medicaid alone. Bed availability for Medicaid-only patients was sometimes contingent on enrollment in a specific managed Medicaid plan. Our results indicate that Californians with Medicaid alone may experience difficulty accessing post-acute SNF care following a hospitalization relative to Californians dually eligible for Medicare and Medicaid. Recent Medicaid budget cuts may lower reimbursement rates and further limit post-acute care access.

